# Metropolitan Workhouse Infirmaries

**Published:** 1890-08-30

**Authors:** Louisa Twining


### Editor's Letter-Box.

[Our Correspondents are reminded that prolixity is a great bar to publication, and that brevity of style and conciseness of statement greatly facilitate early insertion.]

### METROPOLITAN WORKHOUSE INFIRMARIES.

May I be allowed to suggest that the valuable article on this subject in the number of August 23rd should be reprinted separately, and sent to every member of the twenty-four Boards of Guardians in the Metropolitan District? The statement of needed improvements, so clearly defined and suggested (happily being carried out partially by some enlightened Boards), would be the greatest help to those who are trying to press them in other quarters, but who too often find a "Chinese wall" of indifference, aud I may add, of ignorance, to oppose them. If definite proposals could be laid before them, and their practicability proved by actual experience, it would be the greatest possible help in effecting those reforms which so many of us have long desired and urged, and which we may hope shortly to see accomplished.

Louisa Twining.

